# Biosafety Measures at the Dental Office After the Appearance of COVID-19: A Systematic Review

**DOI:** 10.1017/dmp.2020.269

**Published:** 2020-07-27

**Authors:** Fiorella del Pilar Cabrera-Tasayco, Juana Mercedes Rivera-Carhuavilca, Katherine Joselyn Atoche-Socola, Claudio Peña-Soto, Luis Ernesto Arriola-Guillén

**Affiliations:** School of Dentistry, Universidad Científica del Sur, Lima, Peru; Division of Oral Rehabilitation, School of Dentistry, Universidad Científica del Sur, Lima, Peru; Division of Periodontology, School of Dentistry, Universidad Científica del Sur, Lima, Peru; Division of Orthodontics, School of Dentistry, Universidad Científica del Sur, Lima, Peru

**Keywords:** biosecurity, COVID-19, dentistry

## Abstract

The purpose of this research was to determine biosecurity measures at the dental office after the appearance of coronavirus disease 2019 (COVID-19). A search was conducted in the main databases of the scientific literature using the words “COVID-19, coronavirus, SARS-Cov2, biosecurity, disinfection and dentistry.” We analyzed biosecurity and disinfection standards at the dental office and dental health personnel to date, and their adaptation to the needs and way of working of each. As a result, according to the information collected the following procedure was identified: a telephone appointment must be made and a questionnaire should be given before dental care; at arrival to the appointment, the temperature of the patient should be taken and proper cleaning and disinfection of the waiting room should be maintained. Panoramic radiography and CBCT are the auxiliary methods of choice. Absolute isolation and atraumatic restorative therapy techniques are a good alternative to decrease fluid exposure. The removal of protective clothing and accessories must follow a specific order and washing hands before and after is essential. In conclusion, the efficient biosecurity for dentists and patients in all dental care processes before, during, and immediately after the appointment reduces the risk of COVID-19 infection and allows healthy dental care environments.

In December 2019, several cases of pneumonia of unknown origin accompanied by high fever, dry cough, fatigue, and respiratory distress were reported in Wuhan City, China.^1^ This disease was diagnosed as coronavirus disease 2019 (COVID-19) and is caused by severe acute respiratory syndrome coronavirus 2 (SARS-CoV-2) of zoonotic origin (derived from bats). COVID-19 spread rapidly among humans worldwide, leading to its classification as a pandemic by the World Health Organization (WHO) on March 19, 2020.^2,3^ It has been shown that many patients may be carriers of the virus while remaining asymptomatic or presenting only mild symptoms, mainly among children.^4^ The incubation period is between 5 and 14 d, and the elderly and individuals with systemic problems are the most likely to present severe complications.^3^

The contagion can be caused by direct (bodily fluids) or indirect transmission (by contact with surfaces).^5^ The population at large may become infected, however, health professionals and especially dentists are at increased risk due to direct contact with the mouths of their patients and constant exposure to fluids, such as saliva and blood.^6,7^

In March 2020, the WHO published biosecurity measures to be taken by health professionals during patient care, seeking to reduce the high risk to which they are exposed.^8^ In addition, in early April, the American Dental Association (ADA) published guidelines for protective measures in dental offices to maintain biosecurity and thereby minimize the risk of COVID-19 transmission before, during, and after dental care.^9,10^ One of the protective measures recommended is related to the usual clothing of dentists in patient care. Disposable gowns over long-necked jacket and trousers are required. The use of protective lens, respiratory masks such as the N95 or FFP2, face visors, surgical caps, and disposable footwear covers are also recommended for personal and patient protection.^11^ The guidelines also emphasize the need to not leave dental care with protective clothing.^12^

Studies related to biosecurity standards in dental surgery and by health governing entities are needed. Among the standards proposed, a variety of methods of protection and disinfection must be elucidated, taking into account the cost benefits and the essential need for compliance to avoid unwanted contagion. Therefore, this literature review aims to determine the biosecurity measures required in dental offices after the appearance of COVID-19, seeking to provide dental health personnel with updates on the biosecurity and disinfection standards recommended to date, and their adaptation to the needs and ways of working of each.

## METHODS

The bibliographic search carried out included articles published in the Medline databases by means of PubMed, SCOPUS, EBSCO, SCIENCE DIRECT, SCIELO, and LILACS from their beginnings until May 31, 2020, and without language restriction. The keywords used were COVID-19, coronavirus, SARS-Cov-2, biosecurity, disinfection, and dentistry (Table 1). In addition, institutional guides from the WHO and the ADA were taken into account. Observational, descriptive, longitudinal, and systematic review studies were included. In contrast, articles such as letters to the publisher, books, publishers, and case reports were excluded from this review.

**TABLE 1:** Strategies in the Search for Scientific Articles in the Main Sources of Information

Source	Search Terms
PubMed	(((("dentistry"[MeSH Terms] OR "dentistry"[All Fields]) OR "dentistry s"[All Fields]) AND (((((((((([covid 19"[All Fields] OR "covid 2019"[Fields All]) OR "severe acute respiratory syndrome coronavirus 2"[Supplementary Concept]) OR "severe acute respiratory syndrome syndrome 2"[All Fields]) OR "20 19 ncov"[All Fields]) OR "sarscov 2"[All Fields]) OR "2019ncov"[All Fields]) OR ("("wuhan"[All Fields] AND ("coronavirus"[MeSH Terms] OR "coronavirus"[All]Fields)) AND OR ("coronavirus"[All]Fields)) OR "coronaviruses"[All]Fields)) OR "SARS-COV2"[All Fields]
SCOPUS	(TITLE-ABS-KEY (covid-19 ANDTITLE-ABS-KEY (dentistry AND TITLE-ABS-KEY (disinfection))
EBSCO	(coronavirus or covid-19 or 2019-ncov) AND dentistry (coronavirus or covid-19 or 2019-ncov) dental AND
	coronavirus AND (dentistry or dentist or dental) covid-19 AND (dental hygiene or dental care or oral health)
	(dentistry or dentist or dental) AND covid-19 (dentistry or dentist or dental) AND (covid-19 or coronavirus or 2019-ncov)
SCIELO	(covid-19) AND (dentistry) ((covid-19) AND (dentistry)) OR (dental) ((((covid-19) AND (dentistry))) OR (sarscov 2) (coronavirus) AND (dentistry) (covid-19) AND (dental) AND (dental)
LILACS	coronavirus AND dentistry AND (db:("LILACS")) tw:(sarscov 2 dentistry) AND (db:("LILACS")) tw:(coronavirus AND dental) AND (db:("LILACS")) tw:(covid-19 AND dental) AND (db:("LILACS")) tw:(covid-19 AND disinfection AND dental) AND (db:("LILACS"))

On review of the literature, the following biosafety measures were identified.

### Measures Before Dental Care

#### Phone Appointments

All patients requiring dental care must request an appointment by phone in advance. During the call the patient’s risk should be assessed with questions regarding the presence of possible symptoms such as fever, cough, respiratory distress, and if the patient has been any contact with any suspicious or person confirmed as having COVID-19.^13^ Appointments should be staggered to avoid patients accumulating in the waiting room.

#### Waiting Room and Patient Arrival

The whole environment should be considered as being of high risk. Therefore, it is recommended that the dental office provide masks, disinfectant alcohol, and that magazines, ornaments, and objects that may spread the virus contagion be removed.^9,14^ In addition, the temperature of the patient should be taken with a contact-free infrared digital thermometer.^15^ A questionnaire should be given to identify patients with potential COVID-19 infection before dental care (Table 2).

**TABLE 2:** Questionnaire for Patients Before Dental Care

Questions to Be Asked of Patients by Telephone and Face-to-Face
1. Do you have a fever or have you had a fever in the last 14 days?
2. Have you experienced breathing problems such as coughing or shortness of breath in the last 14 days?
3. In the last 14 days have you traveled to countries with documented COVID-19 transmission?
4. Have you been in contact with a patient with suspected or confirmed COVID-19 infection?
5. In the last 14 days have you had close contact with at least 2 people with documented experience of fever or breathing problems?
6. Have you recently participated in a meeting or have you had close contact with many unknown people?

Patients answering “yes” to the questionnaire but without a temperature higher than 98.6°F or 37°C are to be instructed to remain quarantined at home and under surveillance for 14 d. In the case of symptomatic patients, these must promptly contact the nearest health center for evaluation. In the event of any symptoms, the appointment is postponed until after 14 d and the patient is advised to undergo a medical evaluation. Patients answering “yes” to the questionnaire and with a temperature higher than 100.4°F or 38°C should be immediately quarantined and the nearest health center should be contacted. If patients answer “no” to the questionnaire, dental care will proceed.^3,14^

### Biosafety Measures During Dental Care

#### Personal Protection and Transmission Precautions

Dental health personnel must take measures to protect both the patients and themselves. Before any procedure, the clinician must perform hand washing and use different garments to enhance biosecurity in the following order: disposable surgical cap, breathing mask (N95 or FFP2), disposable long-sleeved gown with elasticized wrist cuffs, lenses, facial visor, disposable gloves, and boots.^13,14^

Dental units and work tables must be covered with single-use plastic (film) for each patient.^16^

#### Patient Apparel and Management

The patient should be given a hydrogen peroxide rinse with 1% distilled water to decrease the salivary viral load and should be fitted with disposable boots, a disposable cap, and protective glasses.^6^

#### Instrumental and Clinical Material

Handpieces, micromotors, and ultrasound parts must be disinfected with 96% alcohol, sodium hypochlorite. Rotary systems must have an anti-return system.^9,15^

To minimize the spread of aerosols, a rubber dam should be used in all procedures in addition to performing minimally invasive techniques, such as atraumatic restorative therapy.^17,18^

When auxiliary exams such as X-rays are needed, panoramic X-rays or CT scans are recommended, and if a surgical suture procedure is performed, a resorbable material should be chosen to reduce clinical appointments.^4,6^

In addition to following these measures, a disinfection method should be used for any material extracted from the mouth and sent to the laboratory (eg, prints, bite register, and prosthesis) to prevent cross-contamination.^18,19^

#### Suspected Fluid Exposure

When there is suspicion of exposure, microbiological testing should be performed, and if the diagnosis of COVID-19 is confirmed, the patient should be quarantined and supervised.^20^

### Measures After Dental Care

Following any dental procedure, clothing and accessories should be removed in the following order: disposable surgical gown, gloves, face protector, and finally, the mask. The mask should be removed from the back, without contact with the front. It is recommended to place the mask in a plastic bag and immersed in boiling water for 5 min for proper disinfection. Immersion in sodium hypochlorite can also be used for disinfection purposes.^14,21-23^

#### Cleaning and Disinfection of the Office

It is recommended that the handpiece, micromotor, and any equipment that can be removed from the unit be sterilized and/or autoclaved between each patient, depending on the manufacturer’s specifications, and the same considerations should be taken with nondisposable instruments. In addition, X-ray equipment, lights, and the dental chair must be disinfected according to the manufacturer’s instructions.^24^

Surfaces such as door handles, chairs, desks, elevators, and bathrooms, among others, must be frequently cleaned and disinfected. Disinfectants such as 0.1-0.5% sodium hypochlorite, 62-71% ethanol, or 2% glutaraldehyde can be used for surface decontamination, as well as 62% ethanol or 2% glutaraldehyde in freshly prepared solutions and adequate concentrations.^25^

Protective barriers should be used to cover clinical contact surfaces, especially those that are difficult to clean such as switches on dental chairs, computer equipment, screens. These barriers should be changed between each patient.^26^

#### Waste Management

In relation to the residue discarded, this should be disinfected with a 0.5% sodium hypochlorite solution and then placed in a double-layered bag with a “swan neck” knot which should only be filled to 80% capacity to allow proper closure. Sharp objects should be placed in a double bag.^18,23^

All waste originating from the care process is to be considered as dangerous, and disposable personal protection elements should also be considered as hazardous waste. For biocontaminated waste, red bags should be used, while common waste should be discarded in black bags, and special residue should be placed in yellow bags (Table 3).^27,28^

**TABLE 3:** Waste Management in the Dental Office

Type of Waste	Physical State	Packaging and Disposal	Color
Contact with blood, saliva, aerosols, disposable PPE, gauze, among others	Solid	Plastic bag	Red
Pathological anatomical and non-anatomical waste derived from care	Liquid/solid	Airtight container	Red
Used and unused sharp objects	Solid	Rigid labeled container	Red
Special waste such as unbroken glass, expired dental products and materials, bottled revealing liquids	Solid	Plastic bag	Yellow
Common	Solid	Plastic bag	Black

Abbreviation: PPE, personal protective equipment.

## DISCUSSION

This research was carried out to describe the biosafety guidelines in all dental care processes after the appearance of COVID-19 especially because the full practice of dentistry has been reopened in different cities and because the dental urgencies and emergencies cannot be postponed in most of the cases. Due to the increase in infection worldwide, protective measures have been recommended for health personnel including dentists and dental auxiliary personnel who have more direct contact with patients and require the implementation of protective measures in the dental office before, during and immediately after dental care. Thus, this literature review was performed to evaluate the biosecurity standards published for dental care in the main sources of information in the scientific literature.

Because some people are asymptomatic and do not present specific signs and/or symptoms of the SARS-CoV-2 virus, every patient should be considered as a potential carrier. For this reason, it is necessary for the temperature of the patient to be taken and a questionnaire should be given previously to identify any possible risk factor of the disease. In addition, biosafety protocols should be implemented in dental procedures to reduce the risk of infection.^3,4,14,15^

The protection of dental personnel must be adequately addressed, because this health area is the most exposed to cross-contamination, 1 of these reasons is for the use of dental aerosols (released particles less than 50 microns in diameter) that is produced from dental instruments, such as ultrasonic scalers, air-water syringes, dental handpieces when using rotating systems, which are a source of emission of microorganisms and even droplets (particles smaller in dimension than aerosols) can be produced and could generate a risk of contagion. Even inhaled droplets and aerosol particles have different sites of deposition, inhaled droplets are deposited in the upper regions of the respiratory tract, in contrast, inhaled aerosolized particles can penetrate to the depths of the lungs, where they may be deposited in the alveoli.^29^ Therefore, the use of these systems should be minimized and conventional alternative techniques should be used to reduce bacterial dissemination. In addition, it has been shown that the use of N95 masks is essential for medical personnel. However, daily and continuous use can lead to skin lesions especially on the nose; therefore, it is recommended to optimize their use times among the staff. On the other hand, an alternative to disinfection is the use of ultraviolet light, the use of which, however, is limited due to its high cost.^9,15,30-32^

Surfaces and objects used after care must be constantly disinfected to reduce the risk of cross-contamination. A large number of studies have shown that sodium hypochlorite at 0.1%-0.5%, 62-71% ethanol, and 2% glutaraldehyde are able to disinfect surfaces by decreasing virus load.^24-26,33^ Direct contact with disinfectants, such as alcohol and hypochlorite, may cause skin reactions, such as peeling, cracking, stinging, bleeding, and dermatitis; therefore, the use of protection is recommended for surface disinfection.^31^ In addition, it is important to follow adequate waste management after dental consultation, categorizing the different types of waste into the corresponding packaging, providing better management by the staff responsible for waste disposal.^18,23,27,28^

Moreover, because the appearance of COVID-19 is a recent phenomenon, more research is necessary to clarify the doubts remaining in relation to biosafety for dental offices and procedures and to establish definitive protocols. In the meantime, dentists must reinforce biosafety measures to ensure adequate protection to both the dental professionals and their patients. Therefore, it is of great importance to follow all the stages of structured protocols,^34-36^ such as carrying out a questionnaire and taking the temperature of patients before care, as well as disinfection of waiting rooms and offices, and wearing protective clothing. Moreover, it must be kept in mind at all times that hand washing is essential between each patient. Finally, there must be a protocol of maximum protection that avoids contact with exposed areas after dental care and to perform adequate management of waste after care. Only in this way will the dental care environment have a safe protocol to reduce the risk of infection by this new virus which has led to radical changes worldwide.

## CONCLUSIONS

Efficient biosecurity before, during, and immediately after dental care reduces the risk of COVID-19 infection in dentists and patients and allows greater confidence in the management of the dental environment.
